# Time to focus on the potential benefit of minimally invasive treatment of remnant cystic duct stump stones

**DOI:** 10.1055/a-2505-9123

**Published:** 2025-02-06

**Authors:** Shenggang Zhan, Defeng Li, Yanhui Tian, Ruiyue Shi, Jing Zhou, Lisheng Wang, Jun Yao

**Affiliations:** 1 12387 Department of Gastroenterology, Shenzhen Peopleʼs Hospital, Shenzhen, China

Cystic duct stump stones are a well-known cause of postcholecystectomy syndrome, a term referring to the 10%–30% of patients who complain of upper abdominal pain or jaundice after a cholecystectomy 1 . Until recently, the management of stones in the cystic duct stump has been generally challenging and invasive 2 3 . Herein, we report the minimally invasive and visually successful removal of a cystic duct stump stone using a novel peroral choledochoscope, the Eye-Max (Micro-Tech, China).

A 60-year-old woman complained of having had intermittent right upper abdominal pain for 1 year; 10 years previously she had undergone laparoscopic cholecystectomy. Physical examination was unremarkable other than upper abdominal tenderness. The results of routine laboratory testing were: total bilirubin, 31.6 µmol/L; γ-glutamyl transpeptidase, 136 U/L; alanine aminotransferase, 571 U/L; aspartate aminotransferase, 849 U/L. Magnetic resonance cholangiopancreatography and endoscopic ultrasonography both revealed a stone 6.8 mm × 5.6 mm in size impacted in the remnant cystic duct ( Fig. 1 **a, b** ). With the patient’s consent, it was proposed to remove the stone using a peroral choledochoscope. Biliary cannulation was performed by wire-guided cannulation using a sphincterotome preloaded with a 0.025-inch guidewire (Boston Scientific, USA). The peroral choledochoscope was then advanced into the remnant cystic duct through the working channel of a duodenoscope, and a dark brown stone was seen ( Fig. 1 **c** ). Next, a retrieval basket (Micro-Tech, China) was inserted into the remnant cystic duct through the working channel of the peroral choledochoscope. Finally, the stone was grasped under direct visualization and pulled into the duodenum ( Fig. 1 **d** ). Repeat cholangiography screened visually for further retained stones ( Video 1 ). The patient recovered uneventfully and was discharged 5 days later.

**Fig. 1:**
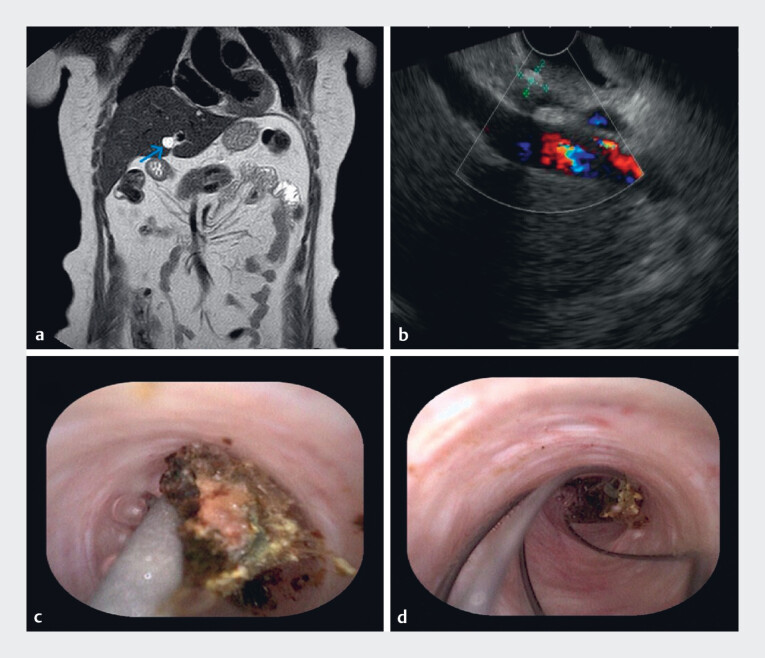
**a** In a 60-year-old woman with a history of intermittent right upper abdominal pain after previous laparoscopic cholecystectomy, magnetic resonance cholangiopancreatography revealed a stone impacted in the remnant cystic duct. **b** Endoscopic ultrasonography also revealed the stone impacted in the remnant cystic duct. **c** A peroral choledochoscope was advanced into the remnant cystic duct and a dark brown stone was seen. **d** The stone was grasped under direct visualization and pulled into the duodenum by a retrieval basket.

Removal of a cystic duct stump stone using a peroral choledochoscope.Video 1

This is the second reported case in which a peroral choledochoscope was used to remove a stone from a cystic duct stump 4 . The peroral choledochoscope has several advantages: minimal invasiveness, low cost, and good visualization. It merits attention now for use in the management of cystic duct stump stones.

Endoscopy_UCTN_Code_TTT_1AR_2AH

## Notes

Endoscopy E-Videos https://eref.thieme.de/e-videos
*E-Videos* is an open access online section of the journal *Endoscopy* , reporting on interesting cases and new techniques in gastroenterological endoscopy. All papers include a high-quality video and are published with a Creative Commons CC-BY license. Endoscopy E-Videos qualify for HINARI discounts and waivers and eligibility is automatically checked during the submission process. We grant 100% waivers to articles whose corresponding authors are based in Group A countries and 50% waivers to those who are based in Group B countries as classified by Research4Life (see: https://www.research4life.org/access/eligibility/ ). This section has its own submission website at https://mc.manuscriptcentral.com/e-videos .
